# COVLIAS 2.0-cXAI: Cloud-Based Explainable Deep Learning System for COVID-19 Lesion Localization in Computed Tomography Scans

**DOI:** 10.3390/diagnostics12061482

**Published:** 2022-06-16

**Authors:** Jasjit S. Suri, Sushant Agarwal, Gian Luca Chabert, Alessandro Carriero, Alessio Paschè, Pietro S. C. Danna, Luca Saba, Armin Mehmedović, Gavino Faa, Inder M. Singh, Monika Turk, Paramjit S. Chadha, Amer M. Johri, Narendra N. Khanna, Sophie Mavrogeni, John R. Laird, Gyan Pareek, Martin Miner, David W. Sobel, Antonella Balestrieri, Petros P. Sfikakis, George Tsoulfas, Athanasios D. Protogerou, Durga Prasanna Misra, Vikas Agarwal, George D. Kitas, Jagjit S. Teji, Mustafa Al-Maini, Surinder K. Dhanjil, Andrew Nicolaides, Aditya Sharma, Vijay Rathore, Mostafa Fatemi, Azra Alizad, Pudukode R. Krishnan, Ferenc Nagy, Zoltan Ruzsa, Mostafa M. Fouda, Subbaram Naidu, Klaudija Viskovic, Mannudeep K. Kalra

**Affiliations:** 1 Stroke Diagnostic and Monitoring Division, AtheroPoint™, Roseville, CA 95661, USA; drindersingh1@gmail.com (I.M.S.); pomchadha@gmail.com (P.S.C.); 2 Advanced Knowledge Engineering Centre, GBTI, Roseville, CA 95661, USA; sushant.ag09@gmail.com; 3 Department of Computer Science Engineering, PSIT, Kanpur 209305, India; 4 Department of Radiology, Azienda Ospedaliero Universitaria (A.O.U.), 09123 Cagliari, Italy; gianchab@yahoo.com (G.L.C.); pascheale@gmail.com (A.P.); psc.dnn@gmail.com (P.S.C.D.); lucasabamd@gmail.com (L.S.); antonellabalestrieri@hotmail.com (A.B.); 5 Department of Radiology, “Maggiore della Carità” Hospital, University of Piemonte Orientale (UPO), Via Solaroli 17, 28100 Novara, Italy; profcarriero@virgilio.it; 6 Department of Radiology, University Hospital for Infectious Diseases, 10000 Zagreb, Croatia; mehmedovic.armin302@gmail.com (A.M.); klaudija.viskovic@bfm.hr (K.V.); 7 Department of Pathology, Azienda Ospedaliero Universitaria (A.O.U.), 09124 Cagliari, Italy; gavinofaa@gmail.com; 8 The Hanse-Wissenschaftskolleg Institute for Advanced Study, 27753 Delmenhorst, Germany; monika.turk84@gmail.com; 9 Department of Medicine, Division of Cardiology, Queen’s University, Kingston, ON K7L 3N6, Canada; johria@queensu.ca; 10 Department of Cardiology, Indraprastha APOLLO Hospitals, New Delhi 110076, India; drnnkhanna@gmail.com; 11 Cardiology Clinic, Onassis Cardiac Surgery Center, 17674 Athens, Greece; soma13@otenet.gr; 12 Heart and Vascular Institute, Adventist Health St. Helena, St. Helena, CA 94574, USA; lairdjr@ah.org; 13 Minimally Invasive Urology Institute, Brown University, Providence, RI 02912, USA; gyan_pareek@brown.edu (G.P.); dwsobel@gmail.com (D.W.S.); 14 Men’s Health Center, Miriam Hospital, Providence, RI 02912, USA; martin_miner@brown.edu; 15 Rheumatology Unit, National Kapodistrian University of Athens, 17674 Athens, Greece; psfikakis@med.uoa.gr; 16 Department of Surgery, Aristoteleion University of Thessaloniki, 54124 Thessaloniki, Greece; tsoulfasg@gmail.com; 17 Cardiovascular Prevention and Research Unit, Department of Pathophysiology, National & Kapodistrian University of Athens, 15772 Athens, Greece; aprotog@med.uoa.gr; 18 Department of Immunology, SGPIMS, Lucknow 226014, India; durgapmisra@gmail.com (D.P.M.); vikasagr@yahoo.com (V.A.); 19 Academic Affairs, Dudley Group NHS Foundation Trust, Dudley DY1 2HQ, UK; george.kitas@nhs.net; 20 Arthritis Research UK Epidemiology Unit, Manchester University, Manchester M13 9PL, UK; 21 Ann and Robert H. Lurie Children’s Hospital of Chicago, Chicago, IL 60611, USA; jsteji1@comcast.net; 22 Allergy, Clinical Immunology and Rheumatology Institute, Toronto, ON M5G 1N8, Canada; almaini@hotmail.com; 23 AtheroPoint LLC., Roseville, CA 95661, USA; surinderdhanjil@gmail.com (S.K.D.); rajvivs888@gmail.com (V.R.); 24 Vascular Screening and Diagnostic Centre, University of Nicosia Medical School, Engomi 2408, Cyprus; anicolaides1@gmail.com; 25 Division of Cardiovascular Medicine, University of Virginia, Charlottesville, VA 22902, USA; as8ah@hscmail.mcc.virginia.edu; 26 Department of Physiology & Biomedical Engineering, Mayo Clinic College of Medicine and Science, Rochester, MN 55905, USA; fatemi.mostafa@mayo.edu; 27 Department of Radiology, Mayo Clinic College of Medicine and Science, Rochester, MN 55905, USA; alizad.azra@mayo.edu; 28 Neurology Department, Fortis Hospital, Bengaluru 560076, India; prkrish12@rediffmail.com; 29 Internal Medicine Department, University of Szeged, 6725 Szeged, Hungary; drnagytfer@hotmail.com; 30 Invasive Cardiology Division, University of Szeged, 1122 Budapest, Hungary; zruzsa@icloud.com; 31 Department of ECE, Idaho State University, Pocatello, ID 83209, USA; mfouda@isu.edu; 32 Electrical Engineering Department, University of Minnesota, Duluth, MN 55812, USA; dsnaidu@d.umn.edu; 33 Department of Radiology, Massachusetts General Hospital, 55 Fruit Street, Boston, MA 02114, USA; mkalra@mgh.harvard.edu

**Keywords:** COVID-19 lesion, lung CT, Hounsfield units, glass ground opacities, hybrid deep learning, explainable AI, segmentation, classification, GRAD-CAM, Grad-CAM++, Score-CAM, FasterScore-CAM

## Abstract

Background: The previous COVID-19 lung diagnosis system lacks both scientific validation and the role of explainable artificial intelligence (AI) for understanding lesion localization. This study presents a cloud-based explainable AI, the “COVLIAS 2.0-cXAI” system using four kinds of class activation maps (CAM) models. Methodology: Our cohort consisted of ~6000 CT slices from two sources (Croatia, 80 COVID-19 patients and Italy, 15 control patients). COVLIAS 2.0-cXAI design consisted of three stages: (i) automated lung segmentation using hybrid deep learning ResNet-UNet model by automatic adjustment of Hounsfield units, hyperparameter optimization, and parallel and distributed training, (ii) classification using three kinds of DenseNet (DN) models (DN-121, DN-169, DN-201), and (iii) validation using four kinds of CAM visualization techniques: gradient-weighted class activation mapping (Grad-CAM), Grad-CAM++, score-weighted CAM (Score-CAM), and FasterScore-CAM. The COVLIAS 2.0-cXAI was validated by three trained senior radiologists for its stability and reliability. The Friedman test was also performed on the scores of the three radiologists. Results: The ResNet-UNet segmentation model resulted in dice similarity of 0.96, Jaccard index of 0.93, a correlation coefficient of 0.99, with a figure-of-merit of 95.99%, while the classifier accuracies for the three DN nets (DN-121, DN-169, and DN-201) were 98%, 98%, and 99% with a loss of ~0.003, ~0.0025, and ~0.002 using 50 epochs, respectively. The mean AUC for all three DN models was 0.99 (p < 0.0001). The COVLIAS 2.0-cXAI showed 80% scans for mean alignment index (MAI) between heatmaps and gold standard, a score of four out of five, establishing the system for clinical settings. Conclusions: The COVLIAS 2.0-cXAI successfully showed a cloud-based explainable AI system for lesion localization in lung CT scans.

## 1. Introduction

COVID-19, the novel coronavirus or SARS-CoV-2, the severe acute respiratory syndrome coronavirus 2, has been a rapidly spreading epidemic that was declared a global pandemic on 11 March 2020 by the World Health Organization (WHO) [1]. As of 20 May 2022, COVID-19 had infected over 521 million people worldwide and has killed nearly 6.2 million [2].

Molecular pathways [3] and imaging [4] of COVID-19 have proven to be worse in individuals with comorbidities such as coronary artery disease [5,6], diabetes [7], atherosclerosis [8], fetal programming [9], pulmonary embolism [10], and stroke [11]. Further, the evidence shows the damage to the aorta’s vasa vasorum, leading to thrombosis and plaque vulnerability [12]. COVID-19 can cause severe lung damage, with abnormalities primarily in the lower region of the lung lobes [13,14,15,16,17,18,19,20]. It is challenging to distinguish COVID-19 pneumonia from interstitial pneumonia or other lung illnesses; as a result, manual classification can be skewed based on radiological expert opinion. As a result, an automated computer-aided diagnostics (CAD) system is sorely needed to categorize and characterize the condition [21], as it delivers excellent performance due to minimal inter-and intra-observer variability.

With the advancements of artificial intelligence (AI) technology [22,23,24], machine learning (ML) and deep learning (DL) approaches have become increasingly popular for detection of pneumonia and its categorization. There have been several innovations in ML and DL frameworks, some of which are applied to lung parenchyma segmentation [25,26,27], pneumonia classification [21,25,28], symptomatic vs. asymptomatic carotid plaque classification [29,30,31,32,33], coronary disease risk stratification [34], cardiovascular/stroke risk stratification [35], classification of Wilson disease vs. controls [36], classification of eye diseases [37], and cancer classification in thyroid [38], liver [39], ovaries [40], prostate [41], and skin [42,43,44]. 

**Figure 1:**
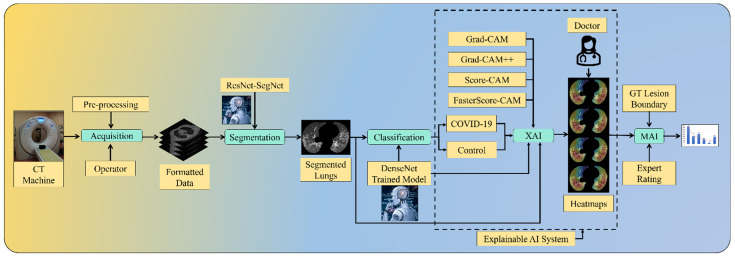
COVLIAS 2.0-cXAI system.

AI can further help in the detection of pneumonia type and can overcome the shortage of specialist personnel by assisting in investigating CT scans [45,46]. One of the key benefits of AI is its ability to emulate manually developed processes. Thus, AI speeds up the process of identifying and diagnosing diseases. On the contrary, the black-box nature of AI offers resistance to usage in clinicians’ settings. Thus, there is a clear need for human readability and interpretability of deep networks, which requires identified lesions to be interpreted and quantified. We, therefore, developed an explainable AI system in a cloud framework, labeled the “COVLIAS 2.0-cXAI” system, which was our primary novelty [47,48,49,50,51,52]. The COVLIAS 2.0-cXAI design consisted of three stages (Figure 1): (i) automated lung segmentation using the hybrid deep learning ResNet-UNet model using automatic adjustment of Hounsfield units [53], hyperparameter optimization [54], and the parallel and distributed nature of design during training; (ii) classification using three kinds of DenseNet (DN) models (DN-121, DN-169, DN-201) [55,56,57,58]; and (iii) scientific validation using four kinds of class activation mapping (CAM) visualization techniques: gradient-weighted class activation mapping (Grad-CAM) [59,60,61,62,63], Grad-CAM++ [64,65,66,67], score-weighted CAM (Score-CAM) [68,69,70], and FasterScore-CAM [71,72]. The COVLIAS 2.0-cXAI was validated by a trained senior radiologist for its stability and reliability. The proposed study also considers different variations in COVID-19 lesions, such as ground-glass opacity (GGO), consolidation, and crazy paving [73,74,75,76,77,78,79,80,81,82]. The COVLIAS 2.0-cXAI design showed the reduction of model size by roughly 30% and an improvement of the online version of the AI system by two times. 

To summarize, our prime contributions in the proposed study consist of six main stages: (i) automated lung segmentation using the HDL-ResNet-UNet model; (ii) classification of COVID-19 vs. controls using three kinds of DenseNets such as DenseNet-121 [55,56,57,83], DenseNet-169, and DenseNet-201; the combination of segmentation and classification depicting the overall performance of the system; (iii) using explainable AI to visualize and validate the prediction of the DenseNet models using four kinds of CAM, namely Grad-CAM, Grad-CAM++, Score-CAM, and FasterScore-CAM, for the first time. This helps us understand the AI model’s learning in the input CT image [35,84,85,86]. (iv) Mean alignment index (MAI) between heatmaps and the gold standard score from three trained senior radiologists, a score of four out of five, establishing the system for clinical applicability. Further, a Friedman statistical test was also conducted to present the statistical significance of the scores from the three experts. (v) Application of the quantization for the trained AI model to make the system light and further ensure faster online prediction. Lastly, (vi) presents an end-to-end cloud-based CT image analysis system, including the CT lung segmentation and COVID-19 intensity map using the four CAM techniques (Figure 1). 

Our study is divided into six sections. The methodology, patient demographics, image acquisition, description of the DenseNet models, and the explainable AI system used in this work are described in Section 2. Section 3 presents the background literature. In Section 4, the models’ findings and their performance evaluation are presented. The discussion and benchmarking sections are in Section 4, and Section 5 presents the conclusions.

## 2. Methodology

### 2.1. Patient Demographics

Two distinct cohorts representing two different countries (Croatia and Italy) were used in the proposed study. The experimental data set included 20 Croatian COVID-19-positive individuals, 17 of whom were male, and the remainder of whom were three females. The GGO, consolidation, and crazy paving had an average value of 4. The second data set included 15 Italian control subjects, ten of whom were male, and the remainder of whom were five females. To confirm the presence of COVID-19 in the selected cohort, an RT-PCR test [87,88,89] was performed for both data sets.

### 2.2. Image Acquisition and Data Preparation

#### 2.2.1. Croatian Data Set

**Figure 2:**
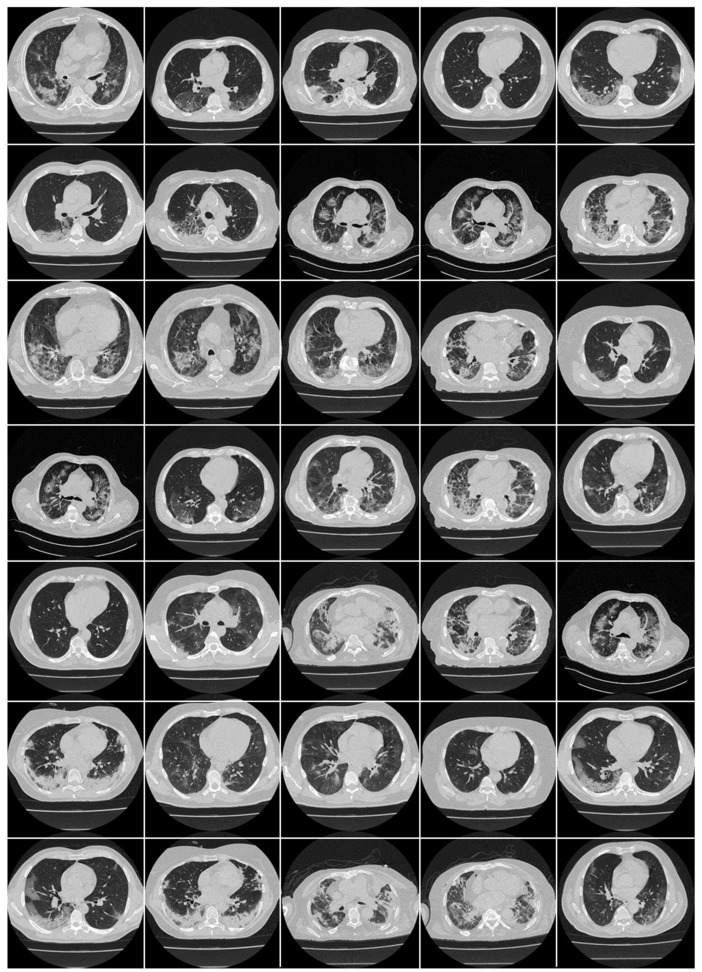
Raw CT slice of COVID-19 patients taken from Croatian data set.

A Croatian data set of 20 COVID-19-positive patients was employed in our investigation (Figure 2). This cohort was acquired between 1 March and 31 December 2020, at the University Hospital for Infectious Diseases (UHID) in Zagreb, Croatia. The patients who underwent thoracic MDCT during their hospital stay showed a positive RT-PCR test for COVID-19 and were also above the age of 18 years. These patients also had hypoxia (oxygen saturation 92%), tachypnea (respiratory rate 22 per minute), tachycardia (pulse rate > 100), and hypotension (systolic blood pressure 100 mmHg). The proposal was approved by the UHID Ethics Committee. The acquisition of the CT data was conducted using a 64-detector FCT Speedia HD scanner (Fujifilm Corporation, Tokyo, Japan, 2017).

#### 2.2.2. Italian Data Set

**Figure 3:**
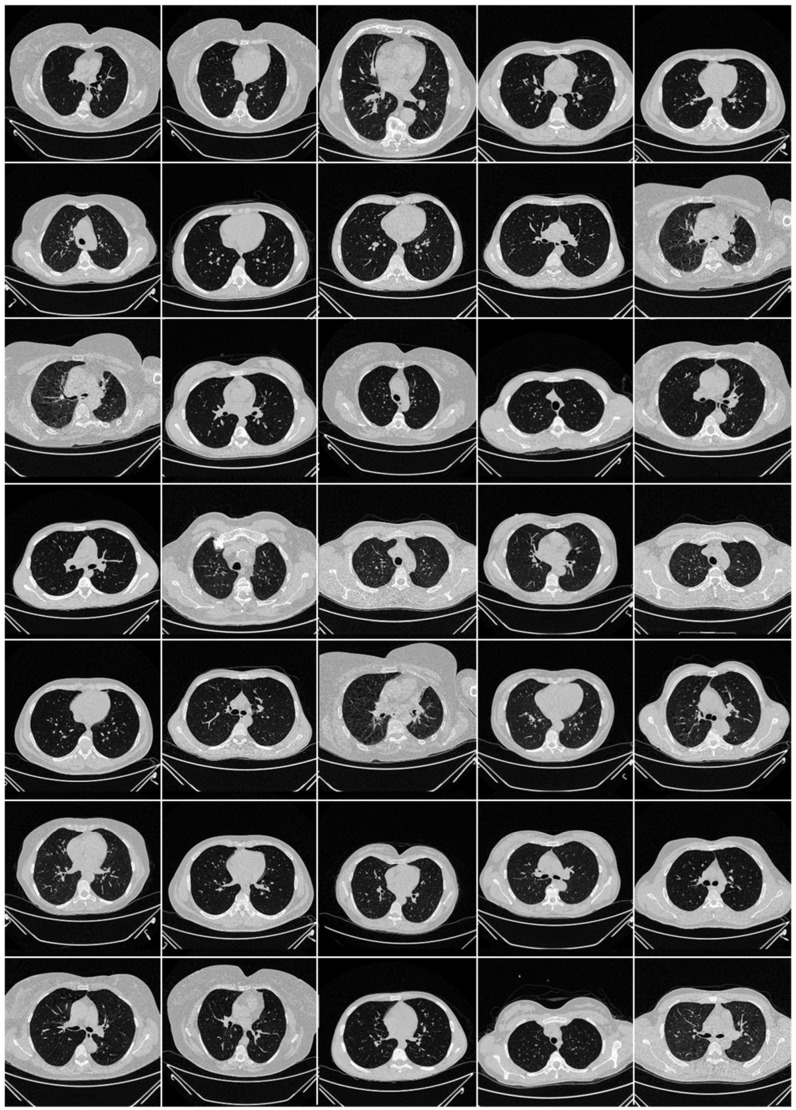
Raw control CT slice taken from Italian data set.

The CT scans for the Italian cohort of 15 patients (Figure 3) were acquired using a 128-slice multidetector-row CT scanner (Philips Ingenuity Core, by Philips Healthcare). The breath-hold procedure was used during acquisition and no contrast agent was administered. To acquire a 1 mm thick slice, a lung kernel of a 768 × 768 matrix together with a soft-tissue kernel was utilized. The CT scans were carried out with a 120 kV, 226 mAs/slice detector configuration (using Philips’ automated tube current modulation—Z-DOM), a spiral pitch factor of 1.08, and a 0.5 s gantry rotation time 64 × 0.625 detector was considered.

### 2.3. Artificial Intelligence Architecture

Recent deep learning developments, such as hybrid deep learning (HDL), have yielded encouraging results [26,27,90,91,92,93,94,95]. We hypothesize that HDL models are superior to SDL models (e.g., UNet [96] and SegNet [97]) due to the joint effect of the two DL models. As a result, we offer a hybrid DL (HDL) such as the ResNet-UNet model that has been trained and tested for the COVID-19-based lung segmentation database in our current study. The aim of the proposed study is directed mainly at the explainable AI (XAI) using the classification models; therefore, we have only used one HDL model. 

#### 2.3.1. ResNet-UNet Architecture

VGGNet [98,99,100] was highly efficient and speedy, but it had a problem with vanishing gradients. During backpropagation, it results in substantially minimal or no weight training because it is multiplied by the gradient at each epoch, and the update is very modest in the initial layers. The residual network, or ResNet [101], was created to solve this problem. Skip connections, a new link, were built into this architecture, allowing gradients to skip a specific set of layers, thus overcoming the problem of vanishing gradient. Furthermore, during the backpropagation step, the local gradient value was preserved by an identity function network. In a ResNet-UNet-based segmentation network, the encoding part of the base UNet network is substituted with ResNet architecture, thus proving a hybrid approach. 

#### 2.3.2. Dense Convolutional Network Architecture

A dense convolutional network (CNN) has an architecture that uses shorter connections across layers, thereby making them highly efficient during training [102]. DenseNet is a CNN where every layer is connected to the ones below it. The primary layer communicates with the 2nd, 3rd, 4th, and so on, whereas the secondary layer communicates with the 3rd, 4th, 5th, and so on. The key idea here was to increase the flow of information between the network layers.

**Figure 4:**
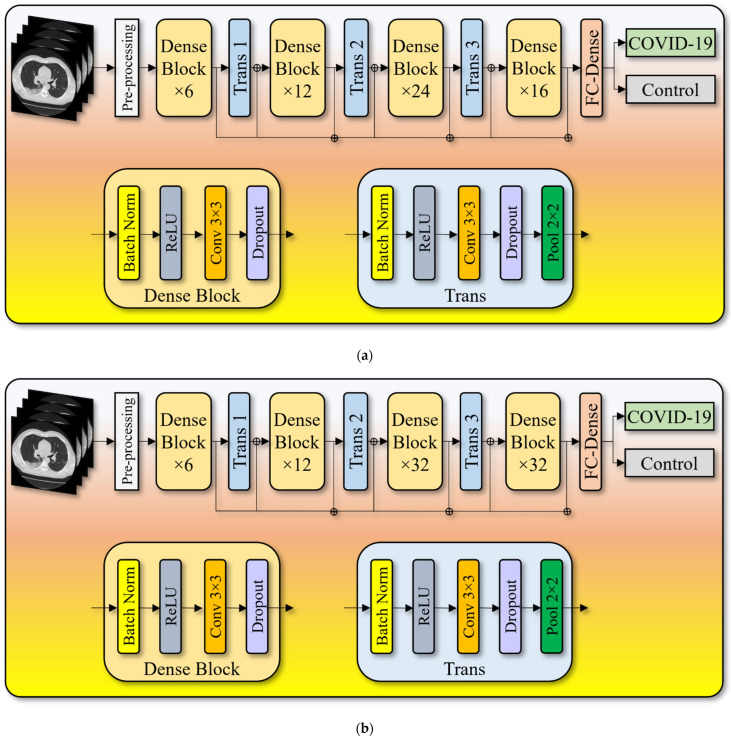

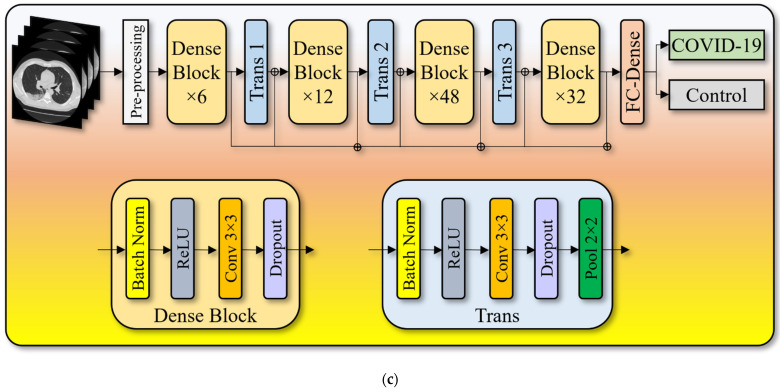
(**a**) DenseNet-121 model. (**b**) DenseNet-169 model. (**c**) DenseNet-201 model.

To maintain the flow of the system, the input received by each layer is forwarded to all the further layers in a feature map. Unlike ResNet, it does not combine features by summarizing them; instead, it concatenates them. As a result, the “jth” layer contains J inputs and comprises feature maps from all the convolutional blocks from the subsequent “J − j” layers that receive their feature maps. Instead of only J connections, the network now has “(J(J + 1))/2” links, like standard deep learning designs. This requires fewer parameters than traditional CNN, avoiding meaningless feature maps to be learned. This paper presents three kinds of DenseNet architectures, namely, (i) DenseNet-121 (Figure 4a), (ii) DenseNet-169 (Figure 4b), and (iii) DenseNet-201 (Figure 4c). Table 1 presents the output feature map sizes of the input layer, convolution layer, dense blocks, transition layers, and fully connected layer followed by the SoftMax classification layer. 

### 2.4. Explainable Artificial Intelligence System for COVID-19 Lesion

We are utilizing machine learning to address more complicated problems as the technology improves and models become more accurate. As machine learning (ML) technology advances, it becomes increasingly sophisticated. This is one of the reasons to use cloud-based explainable AI (cXAI) to help understand how the ML model predicts utilizing a set of tools.

Instead of presenting individual pixels, cXAI is a new approach to displaying attributes that highlight which prominent characteristics of an image had the most significant impact on the model. The effect is seen here (image with heatmap red-yellow-blue), along with which regions contributed to our model’s identification of this image as a husky. Based on the color palette, cXAI highlights the most influential areas in red, the medium influential part in yellow, and the least influential factors in blue. Understanding why a model produced the forecast it did is helpful when debugging a model’s incorrect categorization or determining whether to believe its prediction. Explainability can help (i) debug the AI model, (ii) validate the results, and (iii) provide a visual explanation as to what drove the AI model to classify the image in a certain way. As part of cXAI, we present four cloud-based CAM techniques to visualize the prediction of the AI model and validate it using the color palette as described above. 

#### Four CAM Techniques in Cloud-Based Explainable Artificial Intelligence System

**Figure 5:**
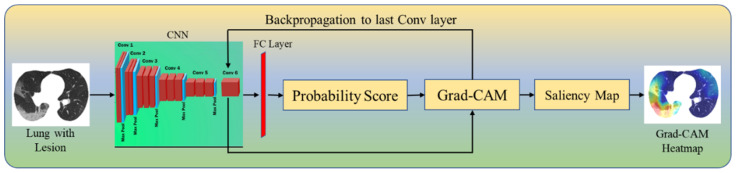
Grad-CAM.

Grad-CAM (Figure 5) generates a localization map that shows the critical places in the image representing the lesions by employing gradients from the target label/class settling into the final convolutional layer. The input image is fed to the model which is then transformed by the Grad-CAM heatmap (Equation (1)) to show the explainable lesions in the COVID-19 CT scans. This image then follows the typical prediction cycle, generating class probability scores before calculating the model loss. Following that, using the output from our desired model layer, we compute the gradient in terms of model loss. Finally, the gradient areas that contribute to the prediction are then preprocessed (Equation (3)), thereby overlaying the heatmap on the original grayscale scans.

**Figure 6:**
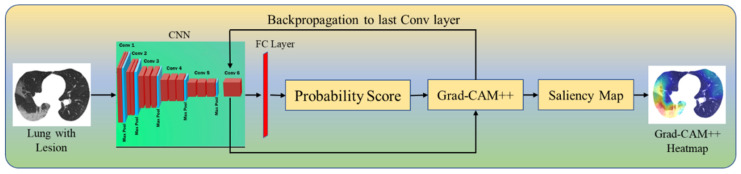
Grad-CAM++.

Grad-CAM++ (Figure 6) is an improved version of Grad-CAM, providing a better understanding by creating an accurate localization map of the identifying object and explaining the same class objects having multiple occurrences. Grad-CAM++ generates a pictorial depiction for the class label as weights derived from the feature map of the CNN layer by considering its positive partial derivatives (Equation (2)). Then, a similar process is followed as in Grad-CAM to produce the gradient’s saliency map (Equation (3)) that contributes to the prediction. This map is then overlaid with the original image.

(1) $$w_{k}^{c}=\frac{1}{Z}\sum_{i}\sum_{j}\left(\frac{\partial Y^{c}}{\partial A_{ij}^{k}}\right)$$

(2) $$w_{k}^{c}=\sum_{i}\sum_{j}a_{ij}^{kc}.\;relu\left(\frac{\partial Y^{c}}{\partial A_{ij}^{k}}\right)\;$$

$$where,\;Y^{c}=\sum_{k}w_{k}^{c}.\sum_{i}\sum_{j}A_{ij}^{k}$$

(3) $$L_{ij}^{c}=\sum_{k}w_{k}^{c}.A_{ij}^{k}\;$$

where $Y^{c}$ represents the final score of class *c* and $A^{k}$ represents the global average pool of the last convolutional layer by considering its linear combination. Estimated weights for the last convolutional layer can be given by $w_{k}^{c}$ for class *c*. $L_{ij}^{c}$ represents a class-specific saliency map for each spatial location (*i*, *j*).

**Figure 7:**
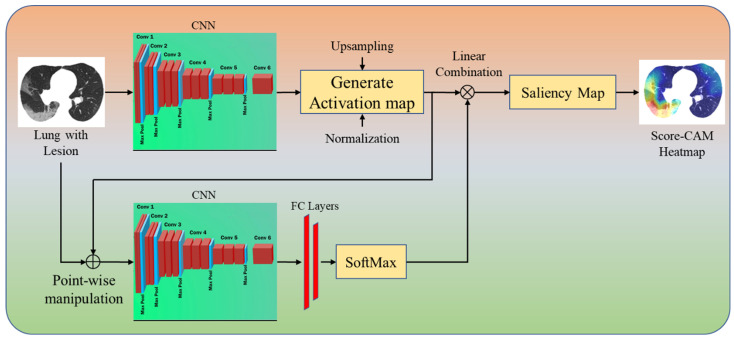
Score-CAM++.

Our third CAM technique is Score-CAM (Figure 7). In this technique, the produced activation mask is used as a mask for the input image, masking sections of the image and causing the model to forecast on the partially masked image. The target class’s score is then used to represent the activation map’s importance. The main difference between Grad-CAM and Score-CAM is that this technique does not incorporate the use of gradients, as the propagated gradients introduce noise and are unstable. The technique is separated into the following parts to obtain the class discriminative saliency map using Score-CAM. (i) Images are processed through the CNN model as a forward pass. The activations are taken from the network’s last convolutional layer after the forward pass. (ii) Each activation map with the shape 1xmxn produced from the previous layer is sampled to the same size as the input image using bilinear interpolation. (iii) The generated activation maps are normalized with each pixel within [0, 1] to maintain the relative intensities between the pixels after upsampling. The formula given in Equation (4) is used for the normalization of the data. (iv) After the activation maps have been normalized, the highlighted areas are projected onto the input space by multiplying each normalized activation map (1 × X × Y) with the original input image (3 × X × Y) to obtain a masked image M with the shape 3 × X × Y (Equation (5)). The resulting masked images M are then fed into a CNN with SoftMax output (Equation (6)). (v) Finally, pixel-wise ReLU (Equation (7)) is applied to the final activation map generated using the sum of all the activation maps for the linear combination of the target class score and each activation map.

(4) $$A_{i,\;j}^{k}=\frac{A_{i,\;j}^{k}}{\max A^{K}-\min A^{K}}$$

(5) $$M^{k}=A^{k}\cdot \;I$$

(6) $$S_{k}=Softmax\;\left(F\left(M^{k}\right)\right)$$

(7) $$L^{c}=ReLU\;\left(\sum_{k}w_{k}^{c}\cdot A^{k}\right)$$

Finally, the fourth technique is labeled FasterScore-CAM. The main innovation of using FasterScore-CAM over the traditional Score-CAM technique is that it eliminates the channels with small variance and only utilizes the activation maps with large variance for heatmap computation and visualization. This selection of activation maps with large variance helps improve the overall speed by nearly ten-fold compared to Score-CAM. 

### 2.5. Loss Function for Artificial-Intelligence-Based Models

During model generation, our system uses the cross-entropy (CE)-loss [103,104,105] function. If CE-loss can be represented by the notation $\alpha_{CE}$, probability of the AI model by $p_{i}$, gold standard label 1 and 0 by g*_i_* and (1 − g*_i_*), respectively, then the loss function equation can be mathematically expressed as shown in Equation (8).

(8) $$\alpha_{\mathrm{CE}}{=-[(g}_{i}{\times \;\log\;p}_{i})+(1\;-\;g_{i}{)\;\times \;\log(1\;-\;p}_{i})]$$

### 2.6. Experimental Protocol 

Our team has demonstrated several cross-validation (CV) protocols using the AI framework; the study uses a standardized five-fold CV technique to train the AI models [106,107]. The data consisted of 80% training data and 20% testing data. K5 CV protocol was adapted where the data were partitioned into five parts, each consisting of a unique training set and testing set and rotated cyclically for all the parts that were used independently. Note that we also used 10% of the data for validation.

The accuracy of the AI system is computed by evaluating the predicted output to the ground-truth label. The output lung mask was just black or white; these measurements were interpreted as binary (1 for white or 0 for black) values. If the symbols TP, TN, FN, and FP represent true positive, true negative, false negative, and false positive, respectively, Equation (9) may be used to evaluate the accuracy of the AI system.

(9) $$\mathrm{Accuracy}\;\left(\%\right)=\left(\frac{\mathrm{TP}+\mathrm{TN}}{\mathrm{TP}+\mathrm{FN}+\mathrm{TN}+\mathrm{FP}}\right)\times 100$$

Precision (Equation (10)) of an AI model is given as the ratio of the correctly labeled classes by the model w.r.t total labels of the COVID-19 class including the false-positive cases. Recall (Equation (11)) of an AI model is given as the ratio of the correctly labeled COVID-19 positive class by the AI model to the total COVID-19 in the data set. F1-score (Equation (12)) is the harmonic average of the precision and recall for the given AI model [108,109,110].

(10) $$\mathrm{Precision}\;=\left(\frac{\mathrm{TP}}{\mathrm{TP}+\mathrm{FP}}\right)$$

(11) $$\mathrm{Recall}\;=\left(\frac{\mathrm{TP}}{\mathrm{TP}+\mathrm{FN}}\right)$$

(12) $$F1-\mathrm{Score}\;=2\times \left(\frac{\mathrm{Recall}\;\times \mathrm{Precision}}{\mathrm{Recall}\;+\mathrm{Precision}}\right)$$

## 3. Results and Performance Evaluation

**Figure A1:**
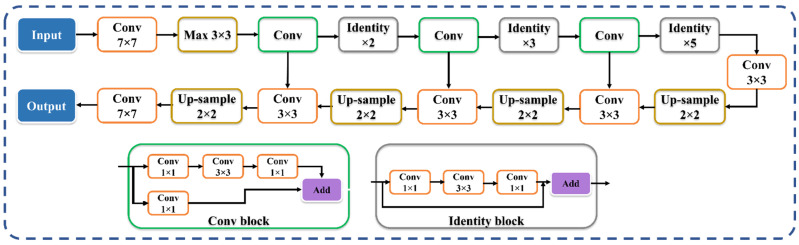
ResNet-UNet architecture.

The proposed study uses the ResNet-UNet model for lung CT segmentation (see Appendix A, Figure A1) and three DenseNet models, namely, DenseNet-121, DenseNet-169, and DenseNet-201 to classify COVID-19 vs. control. The AI classification model was trained on 1400 COVID-19 and 1050 control images, giving an accuracy of 98.21% with an AUC of 0.99 (*p* < 0.0001). 

A confusion matrix (CM) is a table that shows how well a classification model performs on a set of test data for which the real values are known. Table 2 presents CM for three kinds of DenseNet (DN) models (DN-121, DN-169, and DN-201). For DN-121, a total of 1382 and 1020 images were correctly classified and 18 and 30 were misclassified as COVID-19 and control. For DN-169, a total of 1386 and 1028 images were correctly classified and 14 and 22 were misclassified as COVID-19 and control. For DN-201, a total of 1388 and 1038 images were correctly classified and 12 and 12 were misclassified as COVID-19 and control.

### 3.1. Results Using Explainable Artificial Intelligence

#### Visual Results Representing Lesion Using the Four CAM Techniques

**Figure 8:**
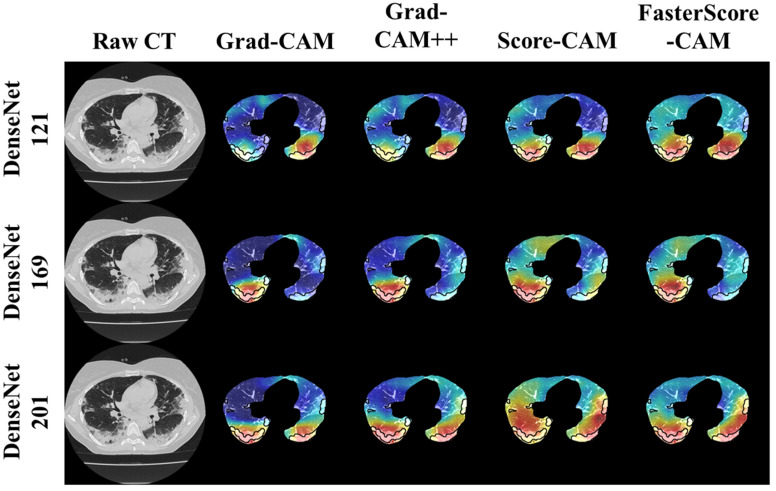
Heatmap using four CAM techniques using three kinds of DenseNet classifiers on COVID-19 lesion images.

The trained classification model from DenseNet-121, DenseNet-169, and DenseNet-201 was taken, and then cXAI was applied to it to generate the heatmap representing the lesion, thereby validating the prediction of the DenseNet models. These images which were used to train the classification models followed the pipeline described in Figure 1, where we first preprocess the CT volume with HU intensities followed by lung segmentation using the ResNet-UNet model. These segmented lung images are then fed to the classification network for the training and application of cXAI. As part of cXAI, we used four CAM techniques, namely, (i) Grad-CAM, (ii) Grad-CAM++, (iii) Score-CAM, and (iv) FasterScore-CAM to visualize the results of the classification model. Figure 8 shows the output from the cXAI, which includes the expert’s lesion localization with black borders, representing the AI model’s missed and correctly captured lesion. 

**Figure 9:**
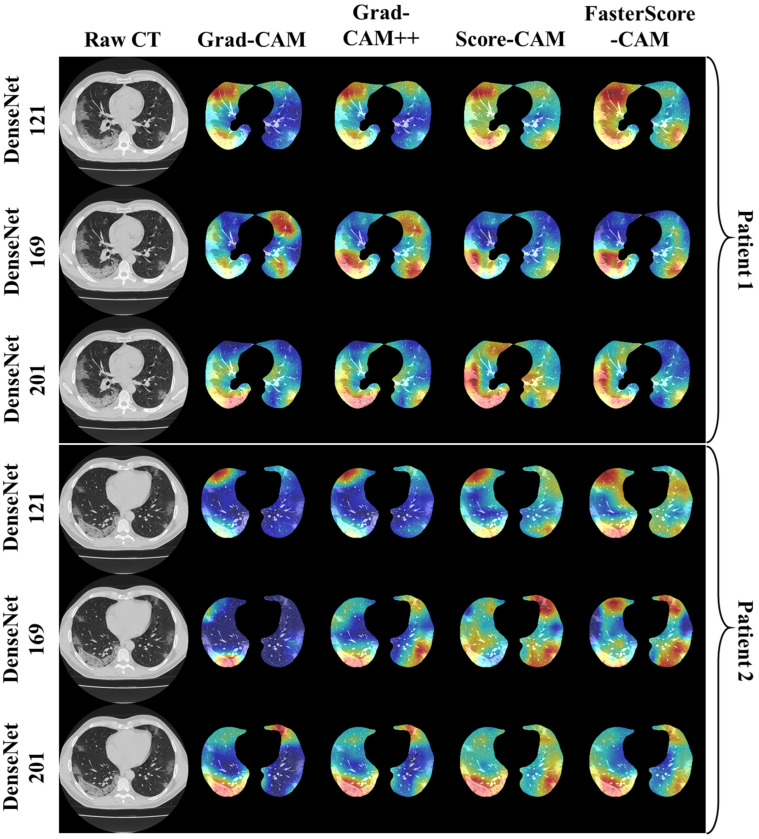
Heatmap using four CAM techniques and three kinds of DenseNet classifiers on COVID-19 lesion images. The top row is the CT slice for patient 1, and the bottom row is the CT slice for patient 2.

**Figure 10:**
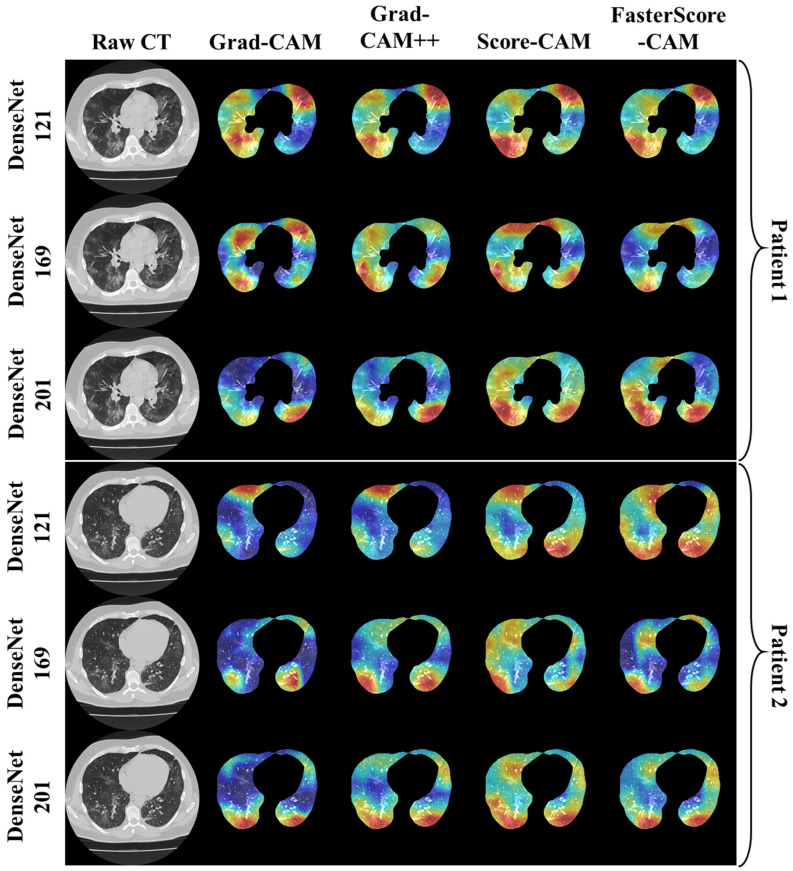
Heatmap using four CAM techniques using three kinds of DenseNet classifiers on COVID-19 lesion images. The top row is the CT slice for patient 1, and the bottom row is the CT slice for patient 2.

**Figure 11:**
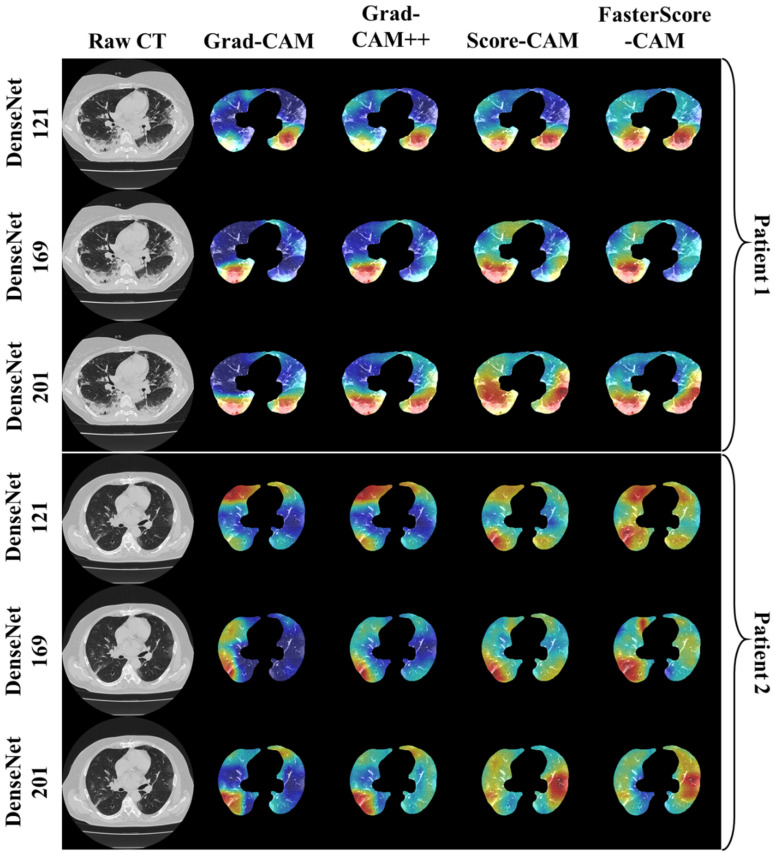
Heatmap using four CAM techniques using three kinds of DenseNet classifiers on COVID-19 lesion images. The top row is the CT slice for patient 1, and the bottom row is the CT slice for patient 2.

**Figure 12:**
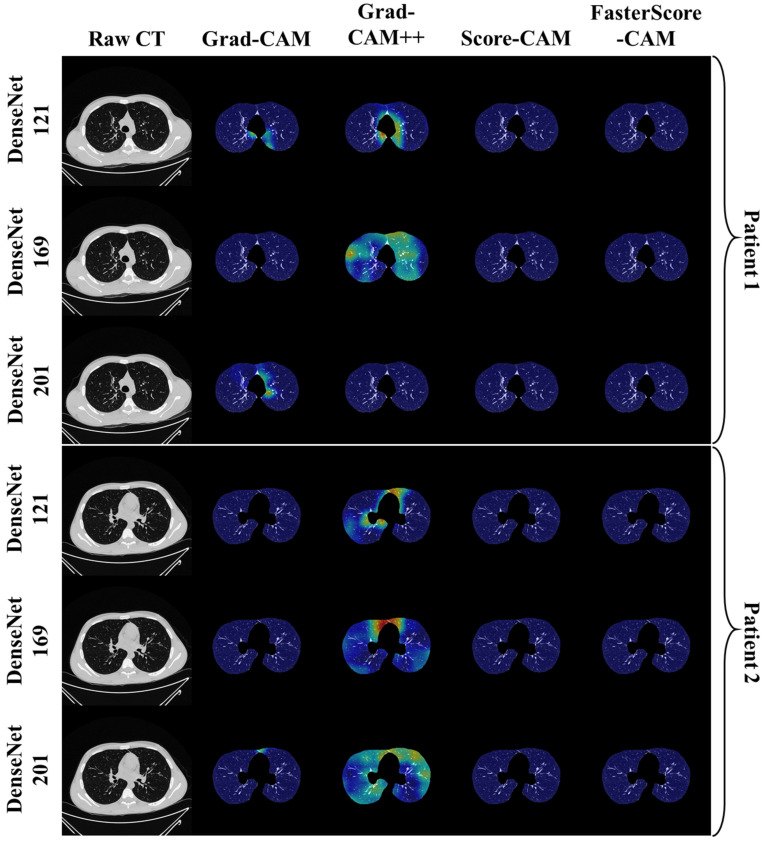
Heatmap using four CAM techniques using three kinds of DenseNet classifiers on control images. The top row is the CT slice for patient 1, and the bottom row is the CT slice for patient 2.

**Figure 13:**
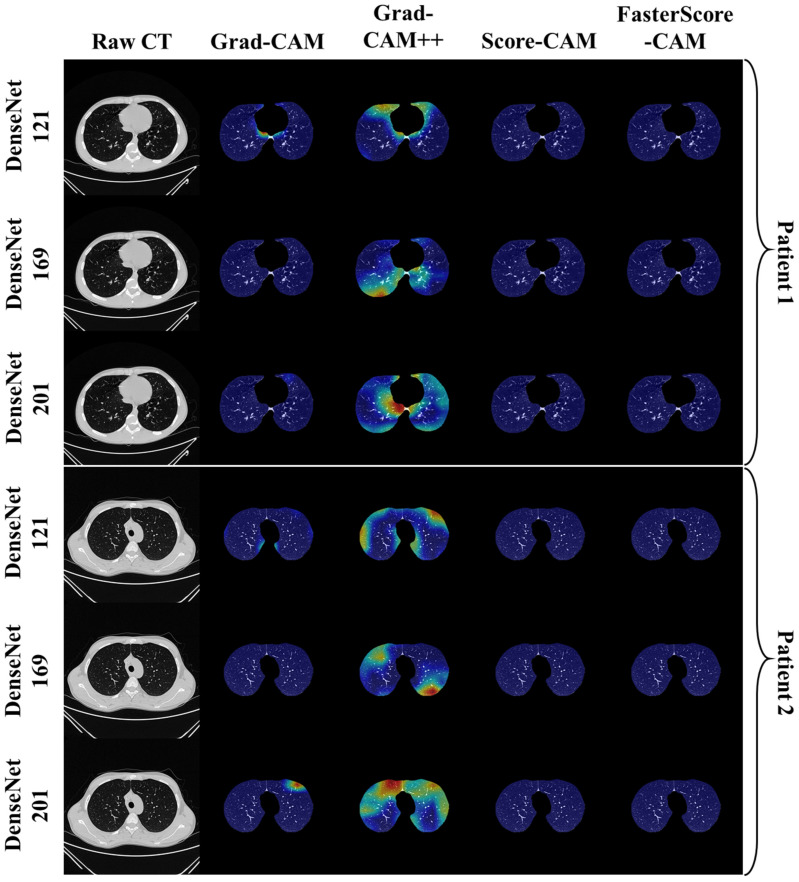
Heatmap using four CAM techniques using three kinds of DenseNet classifiers on control images. The top row is the CT slice for patient 1, and the bottom row is the CT slice for patient 2.

**Figure 14:**
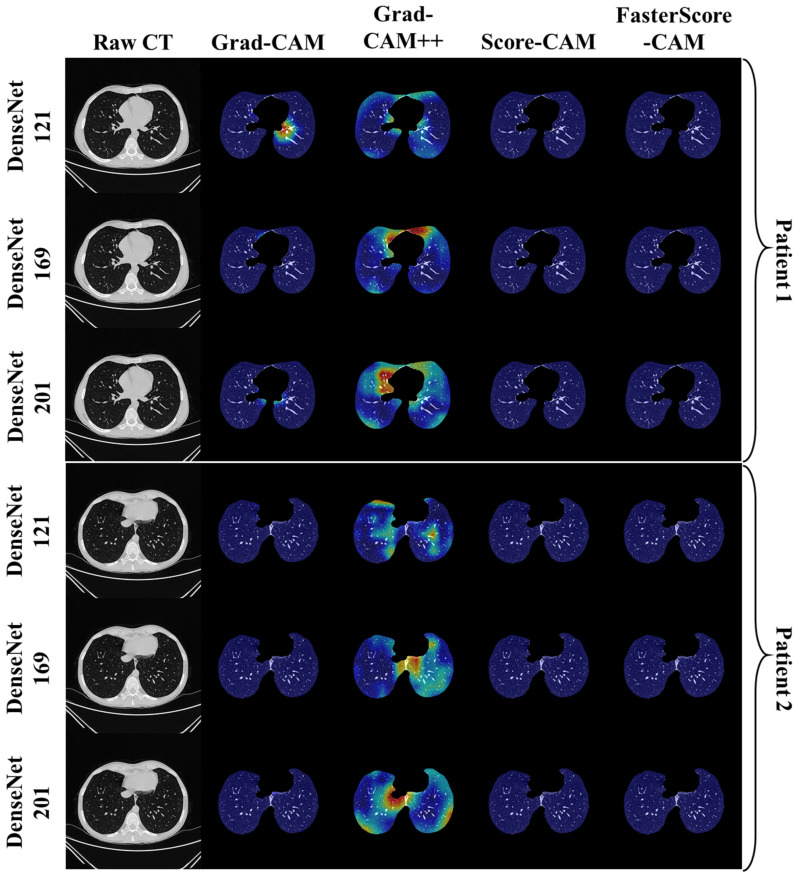
Heatmap using four CAM techniques using three kinds of DenseNet classifiers on control images. The top row is the CT slice for patient 1, and the bottom row is the CT slice for patient 2.

Figure 9, Figure 10, Figure 11, Figure 12, Figure 13 and Figure 14 show the visual results for the three kinds of DenseNet-based classifiers wrapped up with four types of CAM models, namely Grad-CAM (column 2), Grad-CAM++ (column 3), Score-CAM (column 4), and FasterScore-CAM (column 5) on COVID-19 vs. control segmented lung images, where the color map red shows the lesion localization using cXAI, thereby validating the prediction of the DenseNet models. Table 3 presents a comparative analysis of the three DenseNet models used in this study. The performance of the models has been compared using accuracy, loss, specificity, F1-score, recall, precision, and AUC scores. DenseNet-201 is the best-performing model when comparing the accuracy, loss, specificity, F1-score, recall, and precision. However, due to the larger model’s size of 233 MB and a total number of parameters of 203 million, training the batch size of the model was kept at 4. While the batch size while training DenseNet-121 and DenseNet-169 was kept at 16 and 8 due to a smaller model size of 93 MB and 165 MB and further had a lesser number of parameters of 81 million and 143 million, respectively. 

### 3.2. Performance Evaluation

**Figure 15:**
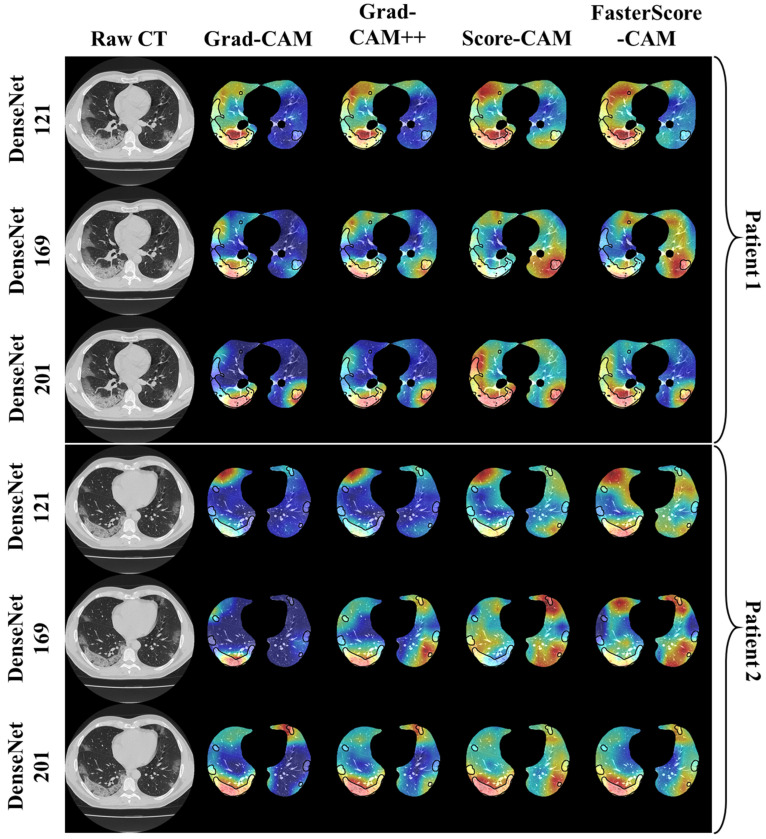
Overlay of ground truth annotation on heatmap using four CAM techniques on three kinds of DenseNet classifiers for COVID-19 lesion images as part of the performance evaluation.

**Figure 16:**
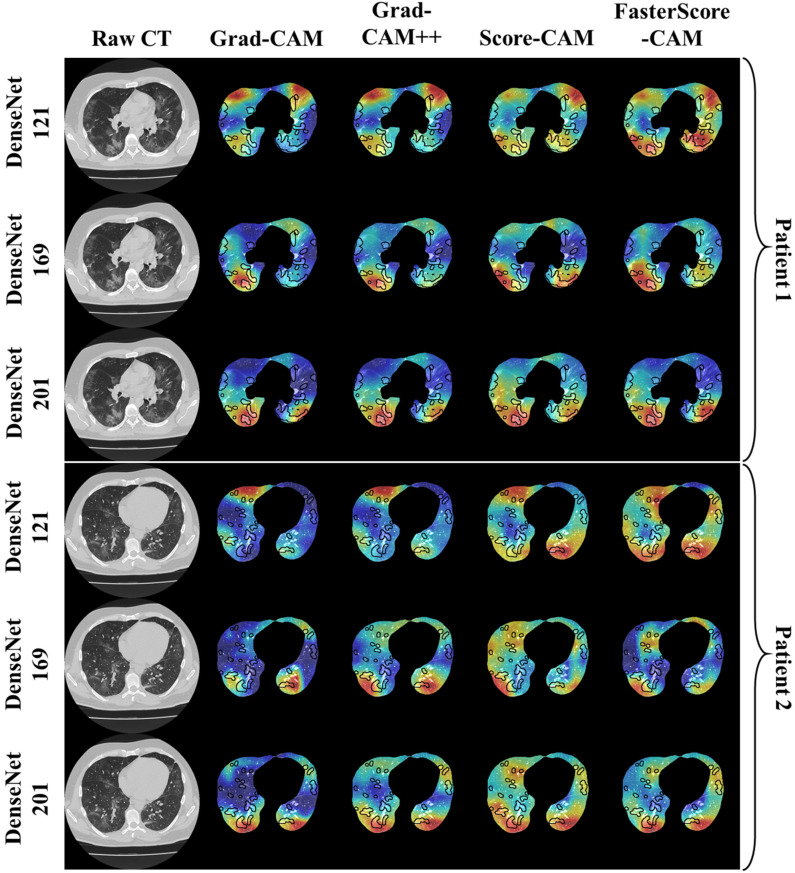
Overlay of ground truth annotation on heatmap using four CAM techniques on three kinds of DenseNet classifiers for COVID-19 lesion images as part of the performance evaluation.

**Figure 17:**
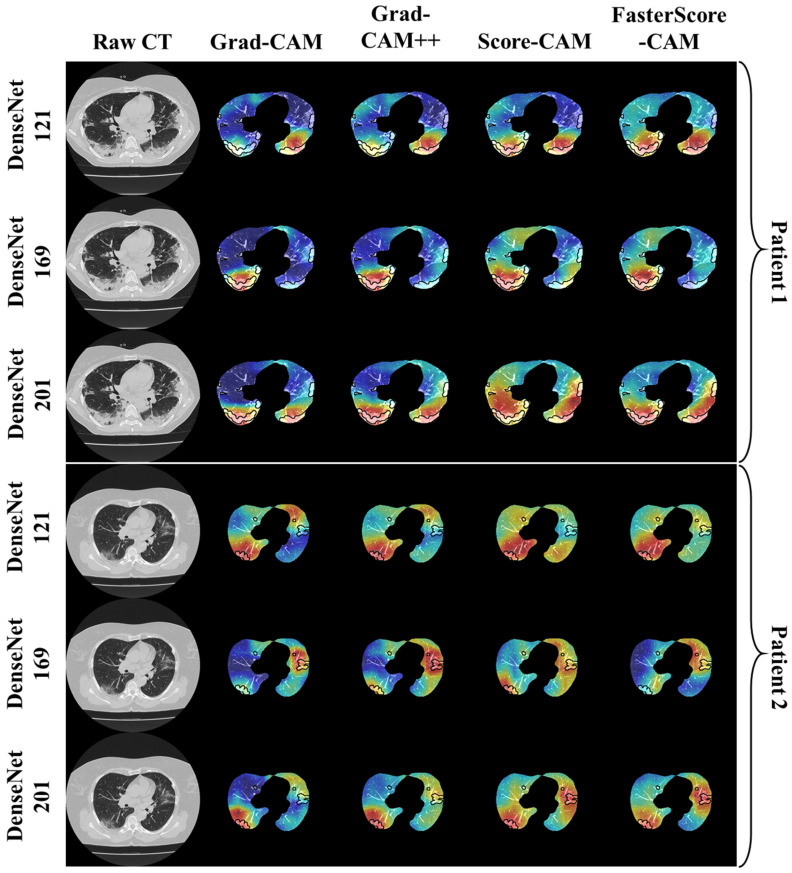
Overlay of ground truth annotation on heatmap using four CAM techniques on three kinds of DenseNet classifiers for COVID-19 lesion images as part of the performance evaluation.

**Figure 18:**
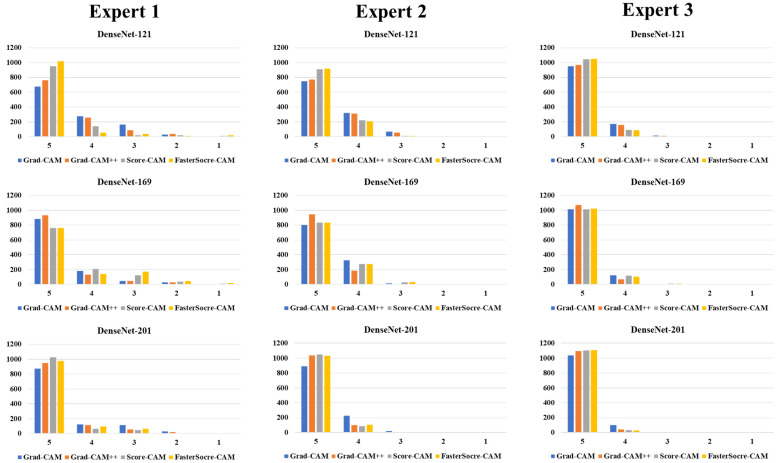
Bar chart representing the MAI.

The proposed study uses two techniques: (i) segmentation of the CT lung; and (ii) classification of the CT lung between COVID-19 vs. controls. For the segmentation part, we have presented mainly five kinds of performance evaluation metrics: (i) area error, (ii) Bland–Altman [111,112], (iii) correlation coefficient [113,114], (iv) dice similarity [115], and (v) Jaccard index. Figure 15, Figure 16 and Figure 17 show the overlay of the ground truth lesions on heatmaps as part of the performance evaluation. The four columns represent Grad-CAM (column 2), Grad-CAM++ (column 3), Score-CAM (column 4), and FasterScore-CAM (column 5) on the segmented lung CT image. For the three DenseNet-based classification models, we introduce a new metric to evaluate the heatmap, i.e., mean alignment index (MAI). This MAI requires grading from a trained radiologist, where the radiologist rates the heatmap image between 1 and 5, with 5 being the best score. This study incorporates inter-observer analysis using three senior trained radiologists from different countries for MAI scoring on the cXAI-generated heatmap of the lesion localization on the images. The scores are then presented in the form of a bar chart (Figure 18) with grading from expert 1 (Figure 18, column 1), expert 2 (Figure 18, column 2), and expert 3 (Figure 18, column 3). 

### 3.3. Statistical Validation

This study uses the Friedman test to prove the statistically significant difference between the means of three or more groups, all of which have the same subjects [116,117,118]. The Friedman test’s null hypothesis states that there are no differences between the sample medians. The null hypothesis will be rejected if the *p*-value calculated is less than the set significance threshold (0.05), and it can be determined that at least two of the sample medians are substantially different from each other. Further analysis of the Friedman test is presented in “Appendix A (Table A1, Table A2 and Table A3)”. It was noted that for all the MAI scores of three experts, the three classification models, namely, DenseNet-121, DenseNet-169, and DenseNet-201, and using the four CAM techniques used in XAI showed significance of *p* < 0.00001. Thus, this proves the reliability of the overall COVLIAS 2.0-cXAI system. 

## 4. Discussion

### 4.1. Study Findings

To summarize, our prime contributions in the proposed study are six types of innovation in the design of COVLIAS 2.0-cXAI: (i) automated HDL lung segmentation using the ResNet-UNet model; (ii) classification of COVID-19 vs. controls using three kinds of DenseNets, namely, DenseNet-121 [55,56,57,83], DenseNet-169, and DenseNet-201; the combination of segmentation and classification improved the overall performance of the system; (iii) using explainable AI to visualize and validate the prediction of the DenseNet models using four kinds of CAM, namely Grad-CAM, Grad-CAM++, Score-CAM, and FasterScore-CAM, for the first time. This helps us understand the AI model’s learning in the input CT image [35,84,85,86]. (iv) Mean alignment index (MAI) between heatmaps and the gold standard score from three trained senior radiologists, a score of four out of five, establishing the system for clinical applicability. Further, a Friedman test was also conducted to present the statistical significance of the scores from the three experts. (v) Application of the quantization to the trained AI model while making the prediction help in faster online prediction. Further, it also reduces the final trained AI model size, making the complete system light. Lastly, (vi) presents an end-to-end cloud-based CT image analysis system, including the CT lung segmentation and COVID-19 intensity map using the four CAM techniques (Figure 1).

**Figure 19:**
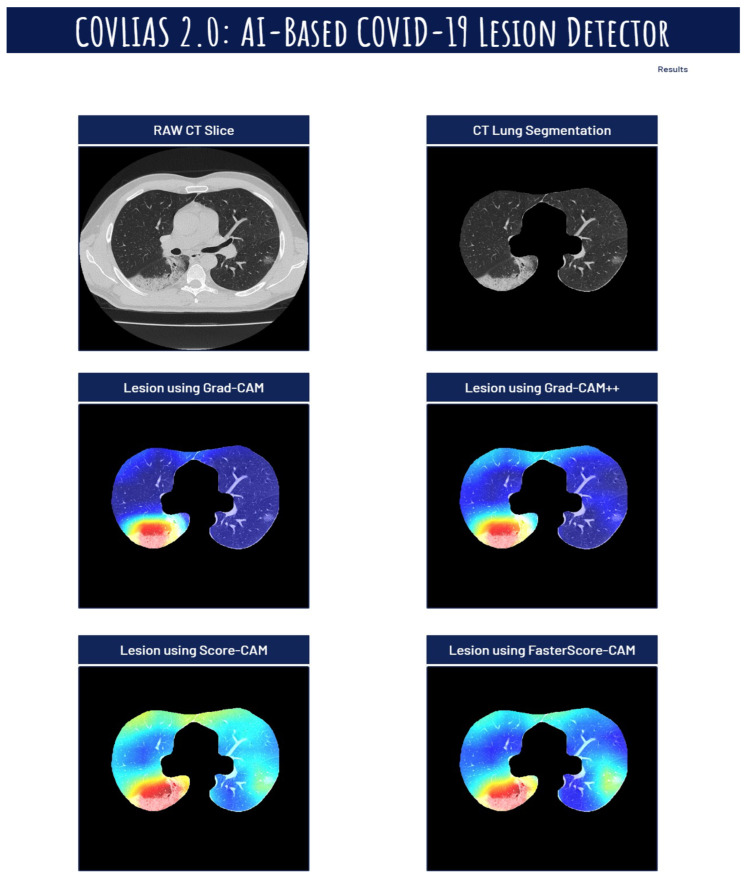
COVLIAS 2.0 cloud-based display of the lesion images using four CAM models.

**Figure 20:**
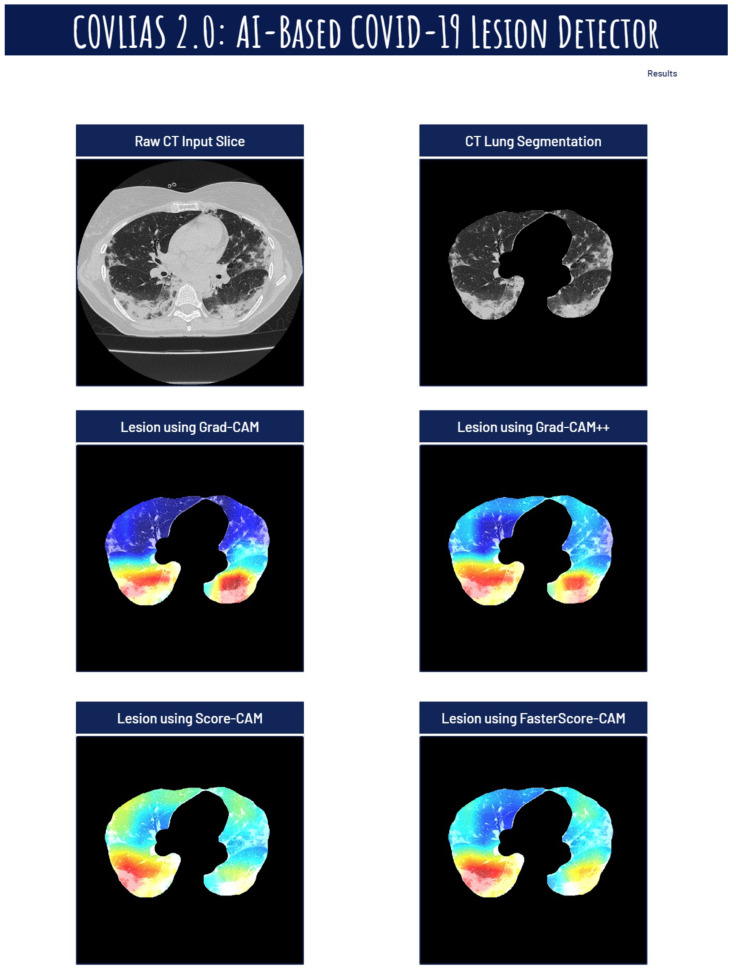
COVLIAS 2.0 cloud-based display of the lesion images using four CAM models.

**Figure 21:**
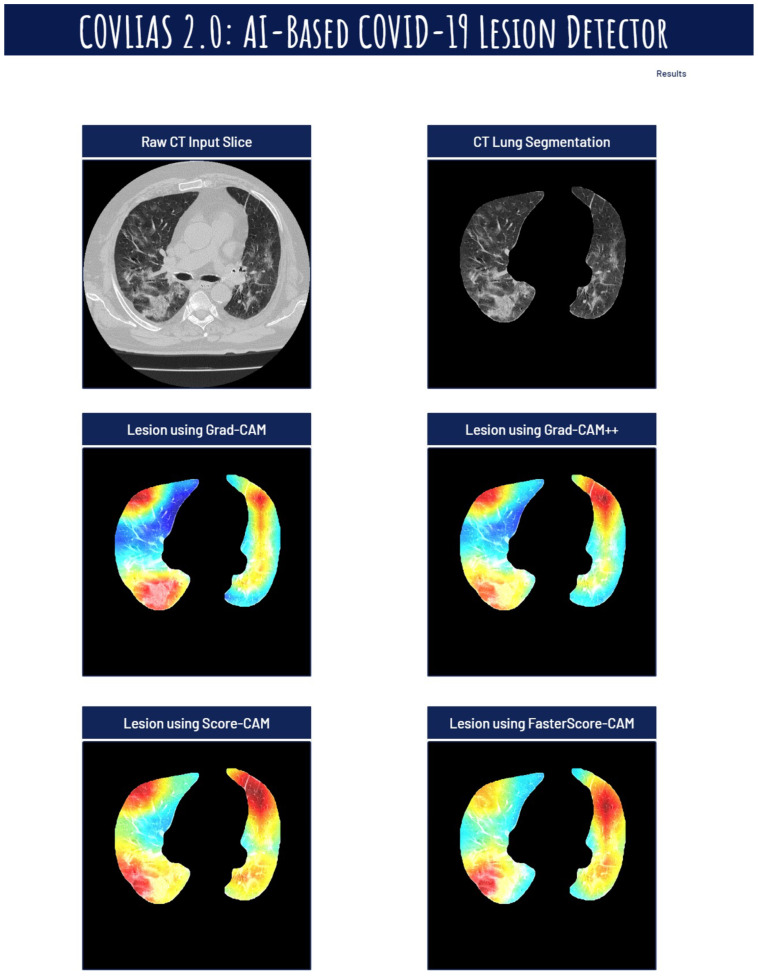
COVLIAS 2.0 cloud-based display of the lesion images using four CAM models.

**Figure 22:**
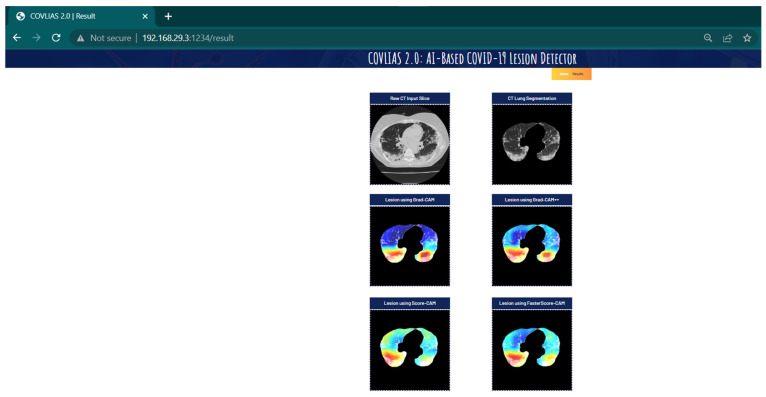
A web-view screenshot.

The proposed study presents heatmaps using four CAM techniques, namely, (i) Grad-CAM, (ii) Grad-CAM++, (iii) Score-CAM, and (iv) FasterScore-CAM. The CT lung segmentation using ResNet-UNet was adapted from our previous publication [93]. This segmented lung is then given as the input to the classification DenseNet models to train in distinguishing between COVID-19-positive and control individuals. The preprocessing involved while training the classification model consists of a Hounsfield unit (HU) adjusted to highlight the lung region (1600, −400), causing the model to train efficiently by improving the visibility of COVID-19 lesions [53]. Further, we have also designed a cloud-based AI system that takes the raw CT slice as the input and then processes this image first for lung segmentation, followed by heatmap visualization using four techniques [119,120,121,122,123]. Figure 19, Figure 20 and Figure 21 represent the output from the cloud-based COVLIAS 2.0-cXAI system (Figure 22, a web-view screenshot). This COVLIAS 2.0-cXAI uses multithreading to process the four CAM techniques in a parallel manner and produces results faster than sequential processing.

While it is intuitive to examine the relationship between demographics and COVID-19 severity [22,124,125,126], it is not always necessarily the case that (i) there can be a relationship between demographics and COVID-19 severity, (ii) there can be data collection with all demographics parameters and COVID-19 severity, (iii) there can be data collection keeping comorbidity in mind, and/or (iv) the cohort sizes are large enough to establish the relationship between demographics and COVID-19 severity. Such conditions are prevalent in our setup and therefore no such relationship could be established; however, as part of the research, one can establish such a relationship along with survival analysis. The objective of this study was squarely not aimed at collecting demographics and relating them to COVID-19 severity; however, we have attempted this in previous studies [127].

Multilabel classification is not new [21,124,128,129]. For multilabel classification, the models are trained with multiple classes, for example, if there are two or more than two classes, then the gold standard must consist of two or more than two classes [124,129]. Note that in our study, the only two classes used were COVID-19 and controls; however, different kinds of lesions can be classified using a multiclass-based classification framework (for example, GGO vs. consolidations vs. crazy paving), which was out of the scope of the current work, but this can be part of the future study. Moreover, inclusion of unsupervised techniques can also be attempted [130].

The total data size for ResNet-UNet-based segmentation was 5000. These trained models were used for segmentation followed by classification on 2450 test CT scans consisting of 1400 COVID-19 cases and 1050 control CT scans. Three kinds of DenseNet classifiers were used for classification of COVID-19 vs. controls. Further, the COVLIAS 2.0-cXAI used the explainable AI using three kinds of Grad-CAM for heatmap generation. Thus, overall, the system used 7450 CT images, which is relatively large. Due to the radiologists’ time and cost reasons, the test data set was nearly 33% of the total data set of the system, which is considered reasonable.

### 4.2. Memorization vs. Generalization for Longitudinal Studies

Generalization is the process where the AI model does not purely depend upon the data sample size for best performance [34,131]. Since the models were trained using K5 cross-validation (CV) protocol (80:20), and the accuracy was predicted on the test data set, which was not part of the training data sets, the process of memorization was thus less likely to happen. Note that for every CV protocol, the “memorization vs. generalization” needs to be evaluated independently, especially keeping the treatment paradigm for longitudinal data sets, which was out of scope for the current settings. From our past experiences, the effect of generalization can be retained in the deep learning framework to a certain degree. In our recent experiments, where we had applied “unseen test data” on our trained AI models, it resulted in encouraging accuracy [27,132], which justifies “superior generalization” in deep learning frameworks, unlike in machine learning frameworks. Since COVLIAS 2.0-cXAI is a deep learning framework, we thus conclude that the cloud-based “COVLIAS 2.0-cXAI” can be adopted for the longitudinal data sets during the monitoring phase.

### 4.3. A Special Note on Training Data Set

We trained the segmentation model using ResNet-UNet on 5000 COVID-19 images. An unseen data set of 2450 (1400 COVID-19 and 1050 control images) was used for testing. Since the training data set was quite large, we did not use augmentation during training protocol. Note that the unseen data (2450) was also not augmented. While several studies have been published that used the augmentation protocol [36,90,94,133,134,135] during classification, our DenseNet models for classification were never modified and never underwent change in rotation, tilt, or orientation. Further, note that we used the DICOM image directly, which contains orientation information. This information was used to solve the problem of rotation, tilting, or any abnormal orientation. This orientation information in the DICOM tag was used to fix the orientation of the image so that the lung is always vertically straight in the image.

### 4.4. A Special Note on Four CAM Models

While DL has demonstrated accuracy in image classification, object recognition, and image segmentation, model interpretability, a key component in model explainability, comprehension, and debugging, is one of the most significant issues. That poses an intriguing question: how can you trust a model’s decisions if you cannot fully justify how it got there? There has been the latest trend in the growth of XAI for a better understanding of the AI black boxes [49,136,137,138,139]. Grad-CAM or Grad-CAM++ produces a coarse clustering map showing the key regions in the picture for predicting any target idea (say, “COVID-19” in a classification network) by using the gradients of any target concept (say, “COVID-19” in a classification network) in the final convolutional layer. In contrast, Score-CAM is built on the idea of perturbation-based approaches that mask portions of the original input and measure the change in target score. The produced activation mask is handled as a mask for the input image, masking sections of the input image and causing the model to predict the partially masked image. The target class score is then used to reflect the significance of the class activation map. While Score-CAM is an excellent method, it, however, takes more time to process compared to other CAM methods. FasterScore-CAM makes Score-CAM more efficient. This was achieved using only the dominating channels with significant variances as the mask image. Thus, a CAM version that is ten times faster than Score-CAM is produced.

### 4.5. Benchmarking the Proposed Model against Previous Strategies

We present the benchmarking strategy in Table 4, and this includes studies that utilized the CAM technique for COVID-19-based lesion localization. Lu et al. [140] presented CGENet, a deep graph model for COVID-19 detection on CT images. First, they established the appropriate backbone network for the CGENet adaptively. The authors then devised a novel graph-embedding mechanism to merge the spatial relationship into the feature vectors. Finally, to improve classification performance, they picked the extreme learning machine (ELM) [24] as the classifier for the proposed CGENet. Based on five-fold cross-validation, the suggested CGENet obtained an average accuracy of 97.78% on a large publicly available COVID-19 data set with ~2400 CT slices. They also compared the performance of CGENet against five existing methods. In addition, based on COVID-19 samples, the Grad-CAM maps were used to offer a visual explanation of CGENet. The authors did not report the AUC values and did not compare the other CAM methods such as Grad-CAM++, Score-CAM, and FasterScore-CAM.

At Tlemcen Hospital in Algeria, Lahsaini et al. [141] first gathered a data set of 4986 COVID and non-COVID images validated by RT-PCR assays. Then, to conduct a comparative analysis, they performed transfer learning on DL models that received the highest results on the ImageNet data set, such as DenseNet-121, DenseNet-201, VGG16, VGG19, Inception Resnet-V2, and Xception [142]. Finally, they proposed an explainable model for detecting COVID-19 in chest CT images and explaining the output decision based on the DenseNet-201 architecture. According to the results of the tests, the proposed design has a 98.8% accuracy rate. It also uses Grad-CAM to provide a visual explanation. The authors did not compare them with other CAM methods such as Grad-CAM++, Score-CAM, and FasterScore-CAM.

Zhang et al. [143] investigated whether combining chest CT and chest X-ray data can assist AI to diagnose more accurately. Approximately 5500 CT slices were collected from 86 participants for this study. The convolutional block attention module was used to create an end-to-end multiple-input deep convolutional attention network (MIDCAN) (CBAM). One of our model’s inputs received a CT picture, while the other received an X-ray image. Grad-CAM was also used to create an explainable heatmap. The suggested MIDCAN had accuracy of 98.02%, sensitivity of 98.1%, and specificity of 97.95%. The authors did not compare the other CAM methods such as Grad-CAM++, Score-CAM, and FasterScore-CAM.

Monta et al. [144] presented the Fused-DenseNet-Tiny, a lightweight DCNN model based on a truncated and concatenated DenseNet. Transfer learning, partial layer freezing, and feature fusion were used to train the model to learn CXR features utilizing 9208 CXR. The proposed model was shown to be 97.99% accurate during testing. Despite its lightweight construction, the Fused-DenseNet-Tiny cannot outperform its larger cousin due to its limited extraction capabilities. The authors also used Grad-CAM to explain the trained AI model visually. The authors did not report the AUC values and did not compare the other CAM methods such as Grad-CAM++, Score-CAM, and FasterScore-CAM.

**Table 4:** Benchmarking table.

C0	C1	C2	C3	C4	C5	C6	C7	C8	C9	C10	C11	C12	C13	C14	C15	C16
SN	Author	Year	TP	TS	IS^2^	TM	DL Model	Modality	XAI	Heatmap Models	AUC	SEN	SPE	PRE	F1	ACC
1	Lu et al. [140]	2021		2482	100 to 500	5	CGENet	CT	✗	Grad-CAM	✗	97.9	97.7	97.7	97.8	97.8
2	Lahsaini et al. [141]	2021	177	4968	✗	6	Transferred DenseNet201	CT	✗	Grad-CAM	0.988	99.5	98.2	97.8	98	98.2
3	Zhang et al. [143]	2021	86	5504	1024(CT) 2048(X-Ray)	8	MIDCAN	CT, X-ray	✗	Grad-CAM	0.98	98.1	98	97.9	98	98
4	Monta et al. [144]	2021		9208	299	7	Fused-DenseNet-Tiny	X-ray	✗	Grad-CAM	✗	✗	✗	98.4	98.3	98
5	Proposed Suri et al.	2022	80	5000	512	3	DenseNet-121 DenseNet-169 DenseNet-201	CT	✓	Grad-CAM Grad-CAM++ Score-CAM FasterScore-CAM	0.99 0.99 0.99	0.96 0.97 0.98	0.975 0.98 0.985	0.96 0.97 0.98	0.96 0.97 0.98	98 98.5 99

TP: total patients; TS: total slice; IS: image size; TM: total models; AUC: area under the curve; SEN (%): sensitivity (or recall); SPE (%): specificity; PRE (%): precision; ACC (%): accuracy.

### 4.6. Strengths, Weakness, and Extensions

The study presented COVLIAS 2.0-cXAI, a cloud-based XAI system for COVID-19 lesion detection and visualization. The cXAI system presented a comparison of four heatmap techniques, (i) Grad-CAM, (ii) Grad-CAM++, (iii) Score-CAM, and (iv) FasterScore-CAM for the first time using three DenseNet models, namely, DenseNet-121, DenseNet-169, and DenseNet-201 for COVID-19 lung CT images. To improve the prediction of the three DenseNet models, we first segment the CT lung using a hybrid DL model ResNet-UNet and then pass it to the classification network. Applying quantization to the three trained AI models, namely, DenseNet-121, DenseNet-169, and DenseNet-201, while making the prediction, helps in faster online prediction. Further, it also reduces the final trained AI model size, making the complete system light. The overall cXAI system incorporates validation of the lesion localization using expert grading, thereby generating an MAI score. Lastly, the study presents an end-to-end cloud-based CT image analysis system (COVLIAS 2.0-cXAI), including the CT lung segmentation (ResNet-UNet) and COVID-19 lesion intensity map using cXAI techniques. This study uses inter-observer variability similar to other variability measurements [145] to score the MAI for lesion localization, which was further validated using the Friedman test.

Even though the three AI models, DenseNet-121, DenseNet-169, and DenseNet-201, produced promising results on a data set from a single location, the study was limited to one observer due to cost, time, and radiologists’ availability. Several kinds of DenseNet systems have been developed which can be tried and the current DenseNet can be replaced by [146,147,148]; as part of the extension to this study, more AI models can be explored and can incorporate the use of the HDL model for binary or multiclass-based classification [128] framework. Explainable AI is an emerging field and many new strategies can be incorporated [47,50,149,150,151,152,153,154,155,156,157]. New techniques have evolved such as SHAP [52,158] and UMAP [159]. Heatmaps produced by Grad-CAM have been used for XAI in several applications [64], where the generated heatmaps are the threshold to compute the lesions which are then compared against the gold standard [49]. Choi et al. [48] used SHAP to demonstrate the high-risk factors responsible for higher phosphate. Further, to improve the speed of the AI model, model optimization techniques such as weight clustering and AI model pruning [160,161,162,163,164] can be applied [115,165,166,167,168,169]. Techniques such as storage reduction are necessary when dealing with AI solutions [51,54,170,171,172]. Fusion of conventional image processing can be used with AI to improve the performance of the system [173,174]. These AI technologies are likely to benefit long-COVID [175].

## 5. Conclusions

The proposed study is the first pilot study that integrates a cloud-based explainable artificial intelligence system using four techniques, namely, (i) Grad-CAM, (ii) Grad-CAM++, (iii) Score-CAM, and (iv) FasterScore-CAM-based lesion localization using three DenseNet models, namely, DenseNet-121, DenseNet-169, and DenseNet-201. Thus, it compares the methods and explainability of the four different CAM strategies for COVID-19-based CT lung lesion localization. DenseNet-121, DenseNet-169, and DenseNet-201 demonstrated an accuracy performance of 98%, 98.5%, and 99%, respectively. The study incorporated a hybrid DL (ResNet-UNet) for COVID-19-based CT lung segmentation using independent cross-validation and performance evaluation schemes. To validate the lesion, three trained senior radiologists scored the lesion localization on the CT lung data set and then compared it against the heatmap generated by cXAI, resulting in the MAI score. Overall, ~80% of CT scans were above an MAI score of four out of five, demonstrating matching lesion locations using cXAI vs. gold standard, thus proving the clinical applicability. Further, the Friedman test was also performed on the MAI scores by comparing the three radiologists. The online cloud-based COVLIAS 2.0-cXAI achieves (i) CT lung image segmentation and (ii) generation of four CAM techniques in less than 10 s for one CT slice. The COVLIAS 2.0-cXAI demonstrated reliability, high accuracy, and clinical stability.

## Figures and Tables

**Table 1:** Output feature map sizes of the three DenseNet architectures.

Layers	Output Feature Size
Input	512 × 512
Conv.	256 × 256
Max Pool	128 × 128
Dense Block 1	128 × 128
Transition Layer 1	128 × 128
	64 × 64
Dense Block 2	64 × 64
Transition Layer 2	64 × 64
	32 × 32
Dense Block 3	32 × 32
Transition Layer 3	32 × 32
	16 × 16
Dense Block 4	16 × 16
Classification Layer (SoftMax)	1024
	2

**Table 2:** Confusion matrix.

DN-121	COVID	Control
COVID	99% (1382)	3% (30)
Control	1% (18)	97% (1020)
DN-169	COVID	Control
COVID	99% (1386)	2% (22)
Control	1% (14)	98% (1028)
DN-201	COVID	Control
COVID	99% (1388)	1% (12)
Control	1% (12)	99% (1038)

**Table 3:** Comparative table for three kinds of DenseNet classifier models.

SN	Attributes	DN-121	DN-169	DN-201
1	# Layers	430	598	710
2	Learning Rate	0.0001	0.0001	0.0001
3	# Epochs	20	20	20
4	Loss	0.003	0.0025	**0.002**
5	ACC	98	98.5	**99**
6	SPE	0.975	0.98	**0.985**
7	F1-Score	0.96	0.97	**0.98**
8	Recall	0.96	0.97	**0.98**
9	Precision	0.96	0.97	**0.98**
10	AUC	0.99	0.99	0.99
11	Size (MB)	93	165	233
12	Batch size	16	8	4
13	Trainable Parameters	80 M	141 M	200 M
14	Total Parameters	81 M	143 M	203 M

DN-121: DenseNet-121; DN-169: DenseNet-169; DN-201: DenseNet-201; # = number of. Bold highlights the superior performance of the DenseNet-201 (DN-201) model.

## Data Availability

Not available.

## Appendix A

**Table A1:** Friedman test using DenseNet-121 model on the MAI score from three experts.

	XAI	Experts	Min.	25th Percentile	Med	75th Percentile	Max	DF-1	DF-2	*p* Value	F
DenseNet-121	Grad-CAM	Expert 1	2	4	5	5	5	2	2278	<0.00001	171.81
		Expert 2	3	4	5	5	5				
		Expert 3	2.7	4.2	4.6	4.8	5				
	Grad-CAM++	Expert 1	2	4	5	5	5	2	2278	<0.00001	244.9
		Expert 2	3	4	5	5	5				
		Expert 3	2.8	4.3	4.6	4.8	5				
	Score-CAM	Expert 1	1	5	5	5	5	2	2278	<0.00001	740.1
		Expert 2	3	5	5	5	5				
		Expert 3	2	4.5	4.7	4.9	5				
	FasterScore-CAM	Expert 1	1	5	5	5	5	2	2278	<0.00001	1072.54
		Expert 2	3	5	5	5	5				
		Expert 3	2.8	4.5	4.7	4.8	5				

Min: minimum; Med: median; Max: maximum; F: Friedman statistics.

**Table A2:** Friedman test using DenseNet-169 model on the MAI score from three experts.

	XAI	Experts	Min.	25th Percentile	Med	75th Percentile	Max	DF-1	DF-2	*p* Value	F
DenseNet-169	Grad-CAM	Expert 1	2	5	5	5	5	2	2278	<0.00001	432.84
		Expert 2	3	4	5	5	5				
		Expert 3	2.7	4.4	4.6	4.8	5				
	Grad-CAM++	Expert 1	2	5	5	5	5	2	2278	<0.00001	689.05
		Expert 2	3	5	5	5	5				
		Expert 3	3.2	4.5	4.7	4.8	5				
	Score-CAM	Expert 1	1	4	5	5	5	2	2278	<0.00001	282.56
		Expert 2	3	4	5	5	5				
		Expert 3	2.8	4.5	4.7	4.8	5				
	FasterScore-CAM	Expert 1	1	4	5	5	5	2	2278	<0.00001	253.15
		Expert 2	3	4	5	5	5				
		Expert 3	2.7	4.4	4.4	4.8	5				

Min: minimum; Med: median; Max: maximum; F: Friedman statistics.

**Table A3:** Friedman test using DenseNet-201 model on the MAI score from three experts.

	XAI	Experts	Min.	25th Percentile	Med	75th Percentile	Max	DF-1	DF-2	*p* Value	F
DenseNet-201	Grad-CAM	Expert 1	2	5	5	5	5	2	2278	<0.00001	499.3
		Expert 2	3	5	5	5	5				
		Expert 3	2.8	4.5	4.7	4.9	5				
	Grad-CAM++	Expert 1	2	5	5	5	5	2	2278	<0.00001	1151.78
		Expert 2	3	5	5	5	5				
		Expert 3	2.7	4.6	4.7	4.9	5				
	Score-CAM	Expert 1	3	5	5	5	5	2	2278	<0.00001	1719.93
		Expert 2	3	5	5	5	5				
		Expert 3	3	4.6	4.7	4.9	5				
	FasterScore-CAM	Expert 1	3	5	5	5	5	2	2278	<0.00001	1239.82
		Expert 2	3	5	5	5	5				
		Expert 3	2.9	4.6	4.7	4.9	5				

Min: minimum; Med: median; Max: maximum; F: Friedman statistics.
